# Trends in HIV counseling and testing uptake among married individuals in Rakai, Uganda

**DOI:** 10.1186/1471-2458-13-618

**Published:** 2013-07-01

**Authors:** Joseph KB Matovu, Julie Denison, Rhoda K Wanyenze, Joseph Ssekasanvu, Fredrick Makumbi, Emilio Ovuga, Nuala McGrath, David Serwadda

**Affiliations:** 1 School of Public Health, Makerere University College of Health Sciences, P.O. Box 7072, Kampala, Uganda; 2 Johns Hopkins Bloomberg School of Public Health, Baltimore, MD, USA; 3 Rakai Health Sciences Program/Uganda Virus Research Institute, P.O. Box 49, Entebbe, Uganda; 4 Gulu University, P.O. Box 166, Gulu, Uganda; 5 University of Southampton, Southampton, UK

**Keywords:** Trends, HCT, Uptake, Married, Couples, Rakai

## Abstract

**Background:**

Despite efforts to promote HIV counseling and testing (HCT) among couples, few couples know their own or their partners’ HIV status. We assessed trends in HCT uptake among married individuals in Rakai district, southwestern Uganda.

**Methods:**

We analysed data for 21,798 married individuals aged 15-49 years who were enrolled into the Rakai Community Cohort Study (RCCS) between 2003 and 2009. Married individuals were interviewed separately but were retrospectively linked to their partners at analysis. All participants had serologic samples obtained for HIV testing, and had the option of receiving HCT together (couples’ HCT) or separately (individual HCT). Individuals were categorized as concordant HIV-positive if both partners had HIV; concordant HIV-negative if both did not have HIV; or HIV-discordant if only one of the partners had HIV. We used *χ*^2^ tests to assess linear trends in individual and couples’ HCT uptake in the entire sample and conducted multinomial logistic regression on a sub-sample of 10,712 individuals to assess relative risk ratios (RRR) and 95% Confidence Intervals (95% CI) associated with individual and couples’ HCT uptake. Analysis was done using STATA version 11.0.

**Results:**

Uptake of couples’ HCT was 27.2% in 2003/04, 25.1% in 2005/06, 28.5% in 2006/08 and 27.8% in 2008/09 (*χ*^2^ for trend = 2.38; *P* = 0.12). Uptake of individual HCT was 57.9% in 2003/04, 60.2% in 2005/06, 54.0% in 2006/08 and 54.4% in 2008/09 (*χ*^2^ for trend = 8.72; *P* = 0.003). The proportion of couples who had never tested increased from 14.9% in 2003/04 to 17.8% in 2008/09 (*χ*^2^ for trend = 18.16; *P* < 0.0001). Uptake of couples’ HCT was significantly associated with prior HCT (Adjusted [Adj.] RRR = 6.80; 95% CI: 5.44, 8.51) and being 25-34 years of age (Adj. RRR = 1.81; 95% CI: 1.32, 2.50). Uptake of individual HCT was significantly associated with prior HCT (Adj. RRR = 6.26; 95% CI: 4.24, 9.24) and the female partner being HIV-positive (Adj. RRR = 2.46; 95% CI: 1.26, 4.80).

**Conclusion:**

Uptake of couples’ HCT remained consistently low (below 30%) over the years, while uptake of individual HCT declined over time. These findings call for innovative strategies to increase demand for couples’ HCT, particularly among younger couples and those with no prior HCT.

## Background

In sub-Saharan Africa, a large proportion of new HIV infections occur within stable relationships [1,2]. A modeled analysis of demographic and health survey data in urban Rwanda and Zambia found that between 55-93% of newly acquired HIV infections among adults occurred within discordant marital or cohabiting relationships [1]. However, several studies have found that over 80% of married couples are not aware of their own or their partner’s HIV status [3,4]. These findings suggest a need for interventions to increase awareness of HIV status and to promote couples’ HIV counseling and testing [5] for the purpose of reducing HIV transmission risk within married couples.

Previous HIV counseling and testing (HCT) efficacy studies suggest that couple counseling and/or partner testing appears to be an effective strategy in altering sexual risk behaviors, especially in HIV-discordant couples [1,6,7]. Dunkle et al. [1] estimated that effective scale-up of programs for voluntary counseling and testing of couples in urban Zambia and Rwanda could reduce heterosexual HIV transmission by 35-80%, assuming an annual incidence of 20% among HIV-discordant couples per year in the absence of an intervention. Another study among HIV-discordant couples in Rwanda, in which both members received couples’ HIV counseling and testing, found that the proportion of discordant couples using condoms increased from 4% to 57% after one year of follow-up [8]. A recent compartmental model to study the effect of HIV status awareness in reducing HIV incidence among married couples suggests that, among HIV-discordant couples, each percentage increase in HIV status awareness reduces HIV incidence by 0.13 and 0.32% for women and men respectively [9]. These findings suggest that interventions aimed at improving couples’ awareness of each other’s HIV status, including couples’ HCT, can contribute to significant reductions in HIV incidence at the population level. Couples’ HCT can also improve identification of previously undiagnosed infections and couple HIV discordance [10] as well as improve timely linkage to HIV care and treatment [11]. Evidence from the HPTN 052 trial found that early treatment initiation, which requires timely linkage to HIV care and treatment, can reduce HIV transmission among discordant couples by 96% [11].

Despite the evidence regarding couples’ HCT effectiveness, uptake remains low (between 5-47%) in most settings [12,13], suggesting a missed opportunity for linking couples to appropriate HIV prevention, care and treatment services. Several factors have been found to hamper effective uptake of couples’ HCT including fear of marital consequences following couples’ HCT [14,15], low male participation [16], and the general perception that monogamy is safe, coupled with beliefs in HIV testing by proxy [17]. Few studies have explored couples’ HIV testing uptake [13,18] but even then, these studies have been conducted in specialized settings (e.g. antenatal clinics or prevention of mother-to-child transmission [PMTCT] sites) and among HIV-discordant couples [19,20] rather than in the general population. Understanding HCT uptake dynamics among couples in a general population context is important in designing effective interventions for HIV prevention at the community level. As access to HIV counseling and testing services increases in sub-Saharan Africa [21], combined with increasing access to antiretroviral therapy; one would expect an increase in the uptake of HCT services among individuals as well as among couples. This would facilitate timely linkage to appropriate HIV prevention, care and treatment services. In this article, we examine trends in voluntary HIV counseling and testing uptake among married individuals enrolled in an ongoing Community Cohort Study in Rakai district, southwestern Uganda.

## Methods

### Study description

The Rakai Community Cohort Study (RCCS) has been described previously [22]. In brief, the Rakai cohort was established in 1994 for a community randomized trial of the control of sexually transmitted diseases (STD) for prevention of HIV (1994-99), and continued annual surveillance thereafter (1999 to-date). The RCCS was conducted in selected study communities that were originally mapped for the STD trial [22], and households within these communities were included in the RCCS. At household level, all consenting adults aged 15-49 years were invited to participate in an interview. Prior to each study visit; a household census was conducted in the 50 study communities to update information on the current residents and to identify eligible persons aged 15-49 years. Participants completed an extensive socio-demographic (age, sex, education, religious affiliation, and type of union, i.e. monogamous or polygamous) and behavioral (engagement in extra-marital relations, lifetime number of sexual partners, prior HIV testing and receipt of HIV test results, ever use of condoms, and HIV risk perception) interviews, administered by trained same-sex interviewers. Venous blood was collected for HIV and STD diagnoses. Serologic diagnosis of HIV was based on two enzyme immunosorbent assays (EIA) (Vironostika HIV; Organon Teknika, Charlotte, North Carolina and Cambridge Biotech, Worcester, Massachusetts, USA) with Western blot confirmation of sero-discordant EIA results and recent HIV sero-converters (HIV WB; Bio-Merieux-Vitek, St Louis, Missiouri, USA).

### The Rakai HCT program

The Rakai HCT program has been described elsewhere [23]. Briefly, all individuals who provided a blood sample at each survey round were asked if they were interested in receiving their HIV test results, and if they were, they were asked if they preferred to receive them together as a couple or individually. On average, HIV test results were available within 1 month from the time of interview and blood draw (rapid HIV testing was not performed). Individuals had the option of receiving their test results and counseling at home (>95% of couples chose to receive HCT at home) or at another venue of their choice. A community-resident HIV counselor (employed by Rakai Health Sciences Program) visited homes of those interested in receiving their HIV test results and provided individual or couples’ HCT based on the couples’ preference. Individuals had the option of declining to receive their HIV test results, despite having initially requested to receive their results, or to receive individual HCT even though they had initially requested to receive them as a couple or the other way round. For each HCT session completed, HIV test counselors completed relevant HCT forms and returned them to the main field office in Kalisizo for entry into the main RCCS database. The HCT forms had provisions for couples that had refused to receive their HIV test results and those who could not be traced in the community either because they out-migrated from the study communities or died between the time of the interview and the delivery of the HIV test results. Individuals who tested HIV-positive were referred to HIV care clinics that exist within the study communities. The clinics, which were established by the Rakai Health Sciences Program, offer antiretroviral therapy, treatment for sexually transmitted infections and other opportunistic infections, prevention of mother-to-child transmission of HIV (PMTCT) services, among others.

The Rakai Health Sciences Program employed trained and certified HIV counselors who were responsible for providing HCT to interested participants. HIV counselors received training in how to conduct couples’ HIV counseling and testing, including how to support HIV-discordant couples, from Ministry of Health trainers. Counselors were also trained in survey research methods to collect basic information about the clients served. The study employed HIV counselors who had been involved in the provision of HCT services in prior studies within the same cohort, so they had the experience needed to provide HCT services to couples and individuals.

HIV test results were provided verbally in a private face-to-face setting in order to ensure privacy and reduce social harm. During the post-test counseling, all study participants were provided with HIV risk-reduction education including faithfulness to one’s spouse (“Be faithful”), and condom use, in line with the National HIV and AIDS Strategic Plan [24]. Information was also provided on couples’ counseling services available, HIV status disclosure, marital stability, nutrition and social support. Participants receiving HCT were helped to assess their own risk for HIV infection and/or transmission and how to reduce this risk. Participants who received individual HCT were encouraged to disclose their HIV test results to their partners, but no involuntary disclosure of HIV status was done since it is not permissible under the National HIV Counseling and Testing Policy [25].

### Measures

As part of the RCCS interviews, participants were asked if they were currently married; the number of marital partners they had and partner identifying information, such as name, household or community of residence, was collected. This information was necessary for linking married individuals to their marital partners at the time of analysis. Married individuals were categorized as being in ‘monogamous relationships’ if they reported only one marital partner or in ‘polygamous relationships’ if they reported more than one marital partner. The definition of a ‘polygamous relationship’ was restricted to the number of marital partners reported by the male partner, since women are culturally not expected to have more than one husband. We used a generic sense of the term “marriage” to encompass all forms of marriage, including cohabitation, those married in church/mosque, and those in traditional and civil marriages. At each visit, married individuals were interviewed separately but at the time of analysis, these individuals were retrospectively linked to their marital partners to form couples, using study identifiers.

A separate couples’ dataset was created for this analysis consisting of HIV status and HCT data as well as information on background socio-demographic and risk behavioral characteristics for married individuals enrolled into the RCCS between 2003 and 2009. Couples in the dataset were considered to be concordant HIV-negative if both partners were HIV-negative (designated as M-F- in this article), concordant HIV-positive if both partners were HIV-positive (M+F+), and HIV-discordant if one of the partners – but not both – was infected with HIV (M-F+ or M+ F-). Uptake of individual HCT was defined as the proportion of couples at each study visit in which one or both members had received individual HCT, and uptake of couples’ HCT was defined as the proportion of couples at each study visit who received HCT, including disclosure of test results, as a couple. Condom use was assessed as use of condoms with any sexual partner in the twelve months preceding the survey, and was coded as Yes = 1 and No = 2. HIV risk perception was categorized as ‘very likely’, ‘somewhat likely’, ‘not likely at all’ or ‘unsure’. The question on HIV risk perception was administered to all participants regardless of their current HIV status, i.e. including those who had previously tested HIV-positive, since interviewers did not know the participants’ HIV status at the time of interview.

For all married individuals in the couples’ dataset, we examined whether they had ever received HCT or disclosed their HIV status to their sexual partners. This question was asked of participants regarding their behaviors prior to 2003/04 and also in between each subsequent RCCS study visit. Assessing prior HCT and disclosure experiences was important because evidence from the Rakai cohort [23] as well as from elsewhere [26] shows that prior receipt of HCT can influence future participation in HCT, including couples’ HCT. In order to identify individuals with prior HCT or HIV status disclosure experience, we linked all individuals in the couples’ dataset to the main RCCS database and checked if one or both partners had received HCT or disclosed their HIV status to their marital partners in the RCCS study visits preceding the current visit. If evidence of prior receipt of HCT was available (regardless of whether one or both partners had ever received HCT), this was categorized as “prior HCT” in the couples’ dataset. Similarly, if evidence of prior HIV status disclosure was available (i.e. ever disclosed to any sexual partner, including the current partner), this was categorized as “prior HIV status disclosure” in the couples’ dataset.

### Statistical analysis

We conducted cross-sectional analyses to assess trends in HIV counseling and testing uptake among 21,798 who participated in four study visits of the Rakai Community Cohort Study between 2003 and 2009 (2003/04; 2005/06; 2006/08; and 2008/09). Couples with complete data were then categorized into those in whom (i) both members had never received HCT (i.e. never tested for HIV), (ii) one or both members had ever received HCT individually, or (iii) both partners had ever received HCT together (i.e., couples’ HCT). Couples were considered to have ever received couples’ HCT if both partners received HCT together in the same sitting, or if the couples were assisted to disclose each other’s HIV test results by a program counselor (i.e. counselor-assisted disclosure). Couples with self-reported HIV status disclosure were excluded from the estimation of couples’ HCT uptake since it was not possible to verify these self-reports. Uptake of couples’ HCT in polygamous relationships was ascertained separately for each husband-wife pair, while keeping the husband constant in each pair. We computed the proportion of couples that had never received HCT, those that had ever received HCT separately and those that had ever received HCT together – overall and by socio-demographic (age, sex, education, religious affiliation and type of union) and risk behavioral (ever use of condoms, lifetime number of sexual partners, engagement in extra-marital relationship, and HIV risk perception) characteristics. We conducted *χ*^2^ tests to assess time trends in HCT uptake for the entire sample and multinomial logistic regression to assess relative risk ratios (RRR) of HCT uptake among a sub-sample 0f 10,712 married individuals who had participated in the RCCS for at least three study visits. The decision on the number of participants to include in the sub-group analysis was based on the need to examine predictors of HCT uptake in individuals who had had a long period of exposure to HCT. The multinomial logistic regression analysis included socio-demographic (age-group, gender and level of education) and behavioral characteristics (extra-marital relations, prior HCT, and couple HIV status) that were found significant (*P* < 0.05) at the bivariate analysis level. Since the primary outcome of the study is uptake of HCT, we used ‘never tested’ as the base outcome. All analyses were adjusted for repeated measures on the same individuals and number of times an individual participated in the study, using robust standard errors. A two-sided p-value of 0.05 or less was regarded as significant. Statistical analyses were done using STATA statistical software, Release 11.0 (College Station, TX: STATA Corporation).

### Ethics statement

The RCCS and the subsequent HCT services were approved by ethics committees at the Uganda Virus Research Institute in Uganda and the Johns Hopkins Bloomberg School of Public Health in the United States of America.

## Results

The trend analysis was conducted with data from 21,798 married individuals (52.4% female) who participated in the RCCS between 2003 and 2009. Of these, 81.6% were in monogamous marital unions while 18.4% were in polygamous marital unions. Of those in polygamous marital unions (n = 4,022), 57.7% were females while 42.3% were males. The total number of participants who were interviewed at each visit was 4,258 in 2003/04; 4,875 in 2005/06; 6,247 in 2006/08 and 6,418 in 2008/09. Forty one per cent of the participants (8,883 of 21,798) reported that they had prior HCT (i.e. individual or couples’ HCT). Overall HIV prevalence was 11.4% (2486 of 21,798).

### Socio-demographic & behavioral characteristics

Table 1 shows socio-demographic and behavioral characteristics of married individuals stratified by sex. The majority of men (85.4-89.6%) were aged 25 years or older (median age = 32 years; interquartile range (IQR): 28-38); had primary or post-primary education (94.9-96.2%) and were in monogamous marital unions (83.1-84.2%). The proportion of men reporting non-marital relations remained high over time (44.4% in 2003/04 and 44.5% in 2008/09) while the proportion of men who perceived themselves to be at high risk of HIV infection significantly increased over time from 3.7% in 2003/04 to 15.2% in 2008/09 (*χ*^2^ for trend = 214.45; *P* < 0.0001). Condom use with any sexual partner in the past year was less stable but did not significantly change from 40% in 2003/04, 37.6% in 2005/06, 42.5% in 2006/08 and 41.5% in 2008/09 (*χ*^2^ for trend = 2.30; *P* = 0.13). The proportion of men who had received prior HCT or had disclosed HIV status to any sexual partner also significantly changed over time with a decline between 2003/04 and 2005/06 followed by an increase between 2006 and 2008.

**Table 1:** Socio-demographic and behavioral characteristics of married individuals in Rakai, Uganda: 2003 – 2009

**Characteristics**	**Males**				**P value (*χ***^**2**^)*	**Females**				**P value (*χ***^**2**^)*
	**2003/04**	**2005/06**	**2006/08**	**2008/09**		**2003/04**	**2005/06**	**2006/08**	**2008/09**	
**All participants**	**1809**	**2383**	**3043**	**3138**		**2449**	**2492**	**3204**	**3280**	
**Socio-demographic characteristics (%)**										
*Age-group*					P < 0.0001					P < 0.0001
15-24	14.6	11.5	11.9	10.3		44.2	38.0	37.0	32.4	
25-34	51.0	50.2	47.4	45.6		42.5	47.5	47.4	49.2	
35+	34.4	38.3	40.8	44.0		13.3	13.3	15.7	18.4	
*Education*					P = 0.364					P = 0.452
No education	4.8	4.4	3.9	3.8		7.9	6.7	7.6	7.0	
Primary	64.3	65.0	66.8	65.4		67.3	66.9	66.3	66.2	
Post-primary	30.9	30.6	29.3	30.8		24.8	26.4	26.2	26.8	
*Type of marital union*					P = 0.731					P = 0.494
Monogamous	83.3	83.1	83.6	84.2		79.0	79.5	79.6	80.5	
Polygamous	16.7	16.9	16.4	15.8		21.0	20.5	20.4	19.5	
**Behavioral characteristics (%)**										
*Non-marital relations in past year*					P = 0.419					P = 0.516
Yes	44.4	44.2	46.2	44.5		3.9	3.5	3.8	3.2	
No	55.6	55.8	53.8	55.5		96.1	96.5	96.2	96.8	
*HIV risk perception*					P < 0.0001					P < 0.0001
Very likely	3.7	4.2	10.1	15.2		3.4	6.3	27.7	32.2	
Somewhat likely	19.6	31.7	46.5	45.1		28.2	27.6	39.3	38.7	
Unlikely	66.6	60.9	36.8	35.1		59.0	53.0	28.2	23.5	
Unsure	10.2	3.2	6.6	4.6		9.4	13.0	4.8	5.6	
*Condom use in past year*					P = 0.001					P < 0.0001
Yes	40.0	37.6	42.8	41.5		20.2	18.9	24.3	25.5	
No	60.0	62.4	57.2	58.5		79.8	81.1	75.7	74.5	
*Prior HCT*					P < 0.0001					P < 0.0001
Yes	56.4	30.2	33.0	35.0		54.8	34.4	40.0	47.6	
No	43.6	69.8	67.0	65.0		45.2	65.6	60.0	52.4	
*Prior HIV status disclosure*					P < 0.0001					P < 0.0001
Yes	50.9	29.1	33.3	35.7		45.8	30.2	36.0	40.3	
No	49.1	70.9	66.7	64.3		54.2	69.8	64.0	59.7	
**HIV status (%)**										
*Individual HIV status*					P = 0.470					P = 0.082
HIV-negative	88.6	89.1	89.3	88.1		87.5	88.6	89.5	88.0	
HIV-positive	11.4	10.9	10.7	11.9		12.5	11.4	10.5	12.0	
*Couple HIV status*					P = 0.305					P = 0.154
M-F-	83.4	84.8	85.2	84.0		84.1	84.8	85.2	84.0	
M + F+	7.6	7.1	6.5	7.9		8.4	7.1	6.4	7.9	
M-F+	5.2	4.3	4.1	4.1		4.1	4.3	4.1	4.2	
M + F-	3.8	3.8	4.2	4.0		3.4	3.8	4.3	4.0	

*Pearson Chi Square test.

The majority of women (81.6-86.7%) were aged between 15-34 years (median age = 27 years; IQR: 22-32), had primary or post-primary education (92.1-93.3%) and were in monogamous marital unions (79-80.5%). As for men, the proportion of women who perceived themselves to be at high risk of HIV infection significantly increased over time, from 3.4% in 2003/04, 6.3% in 2005/06, 27.7% in 2006/08 and 32.2% in 2008/09 (*χ*^2^ for trend = 68.25; *P* < 0.0001). Women’s reported condom use in the past year also increased from 20.2% in 2003/04 and peaked at 25.5% in 2008/09 (*χ*^2^ for trend = 23.32; *P <* 0.0001). The proportion of women who had prior HCT or prior disclosure of their HIV status to any sexual partner also significantly changed with a decline between 2003/04 and 2005/06 followed by an increase between 2006 and 2008.

### HIV prevalence among married individuals

Overall, 11.6% of women and 11.2% of men were HIV-positive. Both men and women experienced a non-significant decline in HIV prevalence between 2003/04 and 2006/08 followed by an increase in 2008/09 (Table 1). The majority of men and women (84.5%) were in concordant HIV-negative (M-F-) marital relationships, 7.3% were in concordant HIV-positive (M + F+) relationships, 4.3% were in HIV-discordant relationships where the female partner was HIV-positive (M-F+) while 3.9% were in HIV-discordant relationships where the male partner was HIV-positive (M + F-). In line with the general HIV prevalence trends, the proportion of men and women in concordant HIV-positive relationships experienced a non-significant decline between 2003/04 and 2006/08 followed by an increase in 2008/09.

### Trends in HIV counseling and testing uptake among married individuals

**Figure 1:**
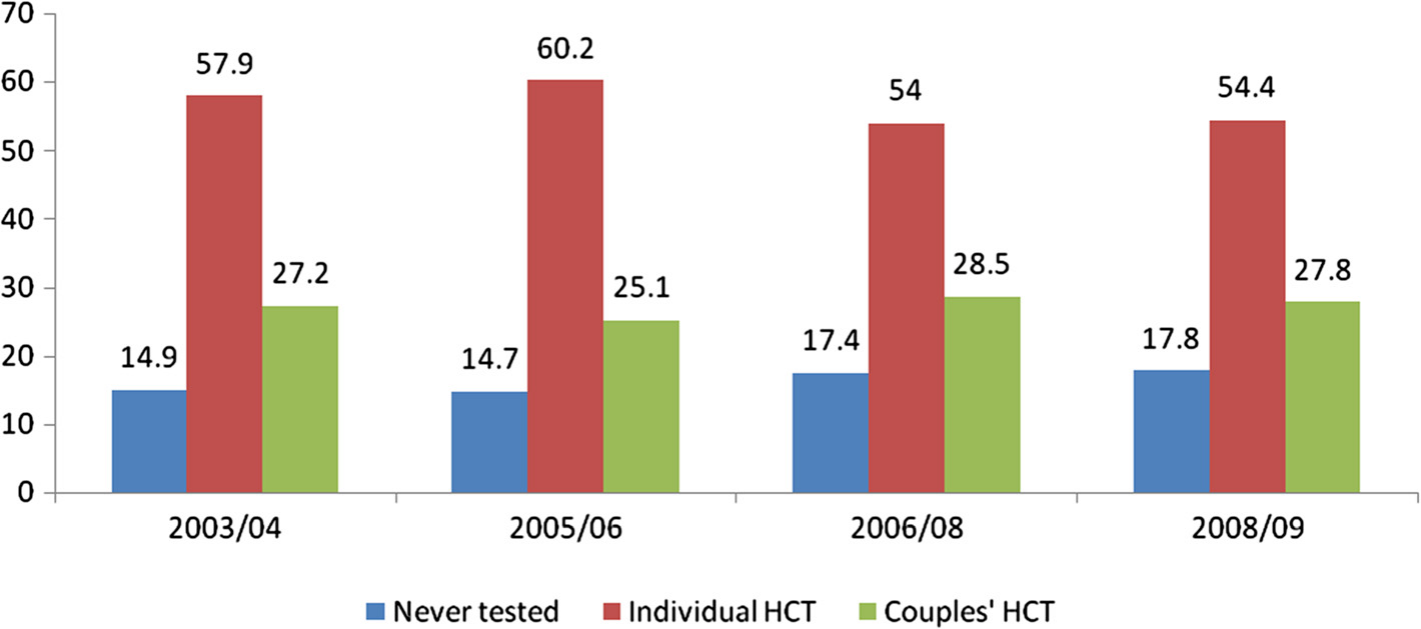
Overall trends in HCT uptake among married individuals in Rakai, Uganda: 2003 – 2009.

Figure 1 shows trends in HCT uptake among married individuals, stratified by those that received HCT as a couple, those that received HCT as individuals (i.e. one or both partners received HCT separately) and those that have never tested for HIV. Overall, uptake of couples’ HCT did not change significantly over time with 27.2% in 2003/04, 25.1% in 2005/06, 28.5% in 2006/08 and 27.8% in 2008/09 (*χ*^2^ for trend = 2.38; *P* = 0.12). Over the same period, the proportion of individuals who received individual HCT significantly declined after an initial increase from 57.9% in 2003/04 to 60.2% in 2005/06 followed by a drop to 54.0% in 2006/08 and 54.4% in 2008/09 (*χ*^2^ for trend = 8.72; *P* = 0.003). The proportion of individuals that have never tested for HIV also significantly increased from 14.9% in 2003/04 and 14.7% in 2005/06 to 17.4% in 2006/08 and 17.8% in 2008/09 (*χ*^2^ for trend = 18.2; *P* < 0.0001) (Figure 1).

#### Trends in HCT uptake by current HIV status

**Figure 2:**
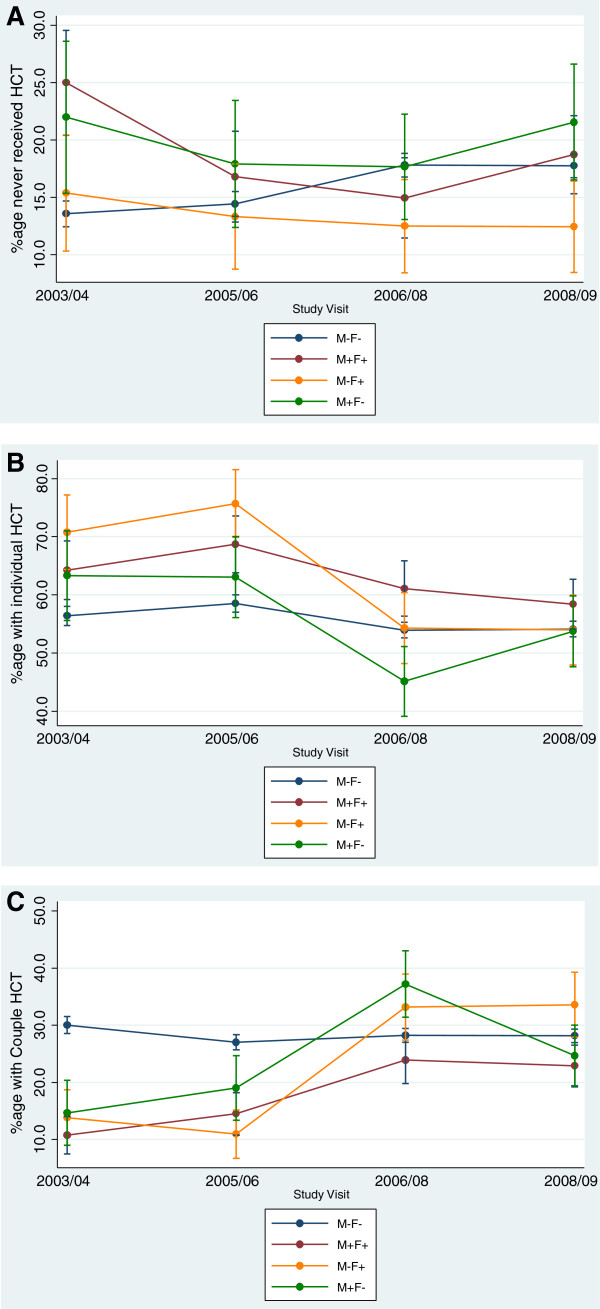
**Trends in HIV counseling and testing among married individuals by current HIV status in Rakai, Uganda: 2003 - 2009. A)** Trends in never-testing among married individuals; **B)** Trends in individual HIV counseling and testing uptake among married individuals; **C)** Trends in couples’ HIV counseling and testing among married individuals.

Figure 2A-C show trends in HCT uptake among married individuals stratified by couple HIV status at the time of enrolment. As shown in Figure 2A, among couples in which both partners were HIV-negative, the proportion of individuals that have never tested for HIV increased significantly from 13.6% in 2003/04 and 14.4% in 2005/06 to 17.8% between 2006-2008 (*χ*^2^ for trend = 28.47; *P* < 0.0001). However, among couples in which both partners were HIV-positive, the proportion of individuals that have never tested for HIV declined from 25% in 2003/04, 16.8% in 2005/06 to 15.0% in 2006/08 but increased slightly to 18.7% in 2008/09 (*χ*^2^ for trend = 241.43; *P* < 0.0001). Among couples where the female partner was HIV-positive (M-F+), the proportion of individuals who had never tested for HIV experienced a non-significant declined from 15.4% in 2003/04 to 13.3% in 2005/06, and remained stable at 12.5% between 2006-2009 (*χ*^2^ for trend = 0.86; *P* < 0.35). In couples where the male partner was HIV-positive (M + F-), the proportion of individuals that had never tested declined from 22.0% in 2003/04 to 17.7% in 2006/08 but increased to 21.6% in 2008/09 (*χ*^2^ for trend = 0.03; *P* < 0.87).

Figure 2B shows trends in individual uptake of HCT, stratified by couple HIV status. In general, uptake of individual HCT increased between 2003 and 2005 but declined between 2006 and 2008. This trend was not quite statistically significant among couples in which both partners were HIV-negative, with the proportion of individual HCT increasing from 56.4% in 2003/04 to 58.5% in 2005/06 and decreasing to 53.9% in 2006/08, and 54.1% in 2008/09 (*χ*^2^ for trend = 3.61; *P* = 0.06). In couples where both partners were HIV-positive, uptake of individual HCT also follows this pattern but is not significant, with 64.2% in 2003/04, 68.7% in 2005/06, 61.1% in 2006/08 and 58.4% in 2008/09 (*χ*^2^ for trend = 1.36; *P* = 0.24). Similar trends were observed among discordant couples, with the only significant decline occurring among M-F + couples for whom individual HCT uptake increased from 70.8% in 2003/05 to 75.7% in 2005/06 but dropped to 54.3% in 2006/08 and 54.0% in 2008/09 (*χ*^2^ for trend = 5.72; *P* = 0.02). However, among M + F- couples, uptake of individual HCT did not significantly change with a decline from 63.3% in 2003/04 to 45.1% in 2006/08 and an increase to 53.7% in 2008/09 (*χ*^2^ for trend = 2.24; *P* = 0.13).

Figure 2C shows trends in couples’ HCT uptake, stratified by couple HIV status. Among couples in which both partners were HIV-negative, uptake of couples’ HCT did not significantly change with 30.0% in 2003/04, 27.0% in 2005/06 and 28.1-28.2% between 2006 and 2009 (*χ*^2^ for trend = 0.82; *P* = 0.36). In couples where both partners were HIV-positive, uptake of couples’ HCT increased significantly from 10.8% in 2003/04, 14.5% in 2005/06 and peaked at 23.9% in 2006/08 before declining slightly to 22.9% in 2008/09 (*χ*^2^ for trend = 17.41; *P* < 0.0001). The trends in uptake of couples’ HCT among discordant couples show a lower uptake between 2003-2005 followed by a sharp increase in uptake between 2006 and 2009. For instance, among M-F + couples, couples’ HCT declined from 13.9% in 2003/04 to 11.0% in 2005/06 but increased sharply to 33.3% in 2006/08 and 33.6% in 2008/09 (*χ*^2^ for trend = 26.27; *P* < 0.0001). In M + F- couples, uptake of couples’ HCT increased from 14.5% in 2003/04 to 19.0% in 2005/06 and peaked at 37.2% in 2006/08 before declining to 24.7% in 2008/09 (*χ*^2^ for trend = 5.72; *P* = 0.02).

#### Trends in HCT uptake by socio-demographic and behavioral characteristics

Table 2 shows trends in non-testing, uptake of individual HCT, and couples’ HCT, by socio-demographic and behavioral characteristics. In terms of non-testing, the proportion of married individuals who have never tested for HIV was significantly higher among participants who were male, those aged 15-24 years, those with primary or post-primary education and those with non-marital relations. For example, in 2008/09, 19.7% of males had never tested compared to 16.9% of females (*P* < 0.0001). Among individuals aged 15-24 years, the proportion of those who have never tested for HIV increased significantly from 16.3% in 2003/04 and 17.1% in 2005/06, to 23.9% in 2006/08 and 29.2% in 2008/09 (*χ*^2^ for trend = 52.03; *P* < 0.0001). The proportion of those who have never tested also significantly increased with higher levels of education. For instance, in 2008/09, the proportion never tested was 9.5% among those with no education; 17.6% among those with primary education and 19.8% among those with post-primary education (*χ*^2^ for trend = 11.45; *P* = 0.0007). The proportion of individuals who have never tested was also consistently and significantly higher among participants reporting non-marital relations compared to those reporting no such relationships. For instance, in 2008/09, 21.6% of those reporting non-marital relations had never tested for HIV compared to 16.6% among those who did not report such relations (P < 0.0001).

**Table 2:** Trends in never-testing (none), individual testing (IHCT) and couples’ testing (CHCT) among married individuals in Rakai, Uganda: 2003 – 2009

**Characteristic**	**2003/04**				**2005/06**				**2006/08**				**2008/09**			
	**N = 4258**				**N = 4875**				**N = 6247**				**N = 6418**			
	**N**	**None %**	**IHCT %**	**CHCT %**	**N**	**None %**	**IHCT %**	**CHCT %**	**N**	**None %**	**IHCT %**	**CHCT %**	**N**	**None %**	**IHCT %**	**CHCT %**
*Gender*																
Male	1809	15.9	55.0	29.2	2383	17.7	56.8	25.6	3043	19.8	50.8	29.4	3138	19.7	51.7	28.6
Female	2449	14.2	60.1	25.7	2492	11.8	63.4	24.8	3204	15.2	57.2	27.7	3280	16.9	57.0	27.1
*Age-group*																
15-24	1347	16.3	61.6	22.0	1221	17.1	63.5	19.4	1547	23.9	55.9	20.2	1385	29.2	55.3	15.5
25-34	1962	13.9	58.2	28.0	2379	13.4	60.4	26.2	2958	15.0	55.4	29.7	3047	15.0	56.4	28.6
35+	949	15.0	52.2	32.9	1275	14.8	56.6	28.6	1742	15.8	50.1	34.0	1986	14.0	50.8	35.2
*Education*																
None	281	10.7	60.9	28.5	272	7.0	64.7	28.3	360	10.0	55.3	34.7	348	9.5	56.9	33.6
Primary	2811	14.7	57.0	28.4	3217	14.3	59.3	26.4	4157	17.0	54.1	28.9	4225	17.6	53.8	28.6
Post-primary	1166	16.5	59.4	24.1	1386	17.1	61.3	21.7	1730	19.9	53.6	26.4	1845	19.8	55.2	25.0
*Non-marital relations*																
Yes	899	19.8	55.4	24.8	1141	20.1	58.8	21.1	1525	22.1	51.6	26.3	1502	21.6	52.7	25.8
No	3359	13.6	58.6	27.8	3734	13.0	60.6	26.4	4722	15.9	54.8	29.3	4916	16.6	55.0	28.4
*HIV risk perception*																
Very likely	151	13.3	65.6	21.2	258	12.8	65.5	21.7	1194	15.5	57.8	26.7	1534	17.2	56.7	26.1
Somewhat likely	1044	16.0	62.7	21.3	1445	17.6	61.3	21.1	2674	17.6	55.5	26.9	2683	18.6	54.5	26.9
Unlikely	2650	14.0	56.2	29.9	2772	13.5	58.8	27.6	2023	18.0	50.7	31.3	1872	17.2	52.5	30.3
Unsure	413	18.6	54.0	27.4	400	13.5	61.8	24.8	356	19.1	49.7	31.2	329	16.7	54.1	29.2
*Prior HCT*																
Yes	2363	n/a	58.9	36.1	1576	n/a	63.6	30.9	2283	n/a	58.3	33.0	2661	n/a	56.8	33.2
No	1895		56.7	16.2	3299		58.5	22.4	3964		51.6	26.0	3757		52.8	24.0
*Prior HIV status disclosure*																
Yes	2043	n/a	55.8	39.7	1446	n/a	60.8	34.7	2167	n/a	57.3	35.3	2442	n/a	56.0	35.6
No	2215		59.9	15.7	3429		59.9	21.1	4080		52.3	25.0	3976		53.5	23.0

In terms of individual HCT, uptake was higher among females than males; participants with prior HCT; those aged 15-24 years; and those that had greater HIV risk perception. In 2008/09, for instance, uptake of individual HCT was 57% among females compared to 51.7% among males (*P* < 0.0001), and 56.8% among those with prior HCT compared to 52.8% among those without prior HCT (*P* = 0.0008). Individual HCT uptake was also consistently higher among younger participants. For instance, in 2008/09, the proportion who received individual HCT was 55.3% among 15-24 year olds compared to 50.8% among those aged 35 years or more (*P* = 0.01). Table 2 also shows that uptake of individual HCT varies with changing levels of HIV risk perception, and this was observed between groups and across the years: for instance, in 2006/08, 57.8% of participants who felt they were very likely to be at risk of HIV infection received individual HCT compared to 55.5% who felt they were somewhat likely to be at risk, 50.7% who felt they were unlikely to be at risk, and 49.7% who were unsure of their risk (*χ*^2^ for trend = 5.84; *P* = 0.01).

Uptake of couples’ HCT was higher in men than women, as well as among older and less educated participants and participants with prior HCT and disclosure experiences. Uptake of couples’ HCT also consistently increased with age between groups and over the years with the 15.5% of 15-24 years old, compare to 28.6% of 25-34 year olds and 35.2% of 35 plus years undergoing HIV testing in 2008/09 (*χ*^2^ for trend = 87.16; *P* < 0.0001). In contrast, couples’ HCT decreased with increasing education. For instance, in 2008/09, the proportion of individuals who received couples’ HCT was 33.6% among those with no education, 28.6% among those with primary education and 25% among those with post-primary education (*χ*^2^ for trend = 7.98; *P* = 0.005). Uptake of couples’ HCT was also lower in those reporting non-marital relationships (e.g. in 2005/06, 21.1% among those with non-marital relations vs. 26.4% among those without non-marital relations, *P* = 0.0003), and higher in those who had prior HCT. In 2008/09, for instance, 33.2% of participants with prior HCT received couples’ HCT compared to 24% among those without prior HCT (*P* < 0.0001). Similarly, couples’ HCT uptake was consistently higher in those with a history of HIV status disclosure to any sexual partner, including the current partner. In 2008/09, 36% of those who reported prior HIV status disclosure received couples’ HCT compared to 23% among those with no history of HIV status disclosure (*P* < 0.0001).

### Predictors of HIV counseling and testing uptake among married individuals in Rakai, Uganda

We conducted a sub-group analysis among 10,712 individuals (49.1% of the original sample) who participated in the RCCS for at least three study visits to assess the predictors of individual and couples’ HCT uptake. Table 3 shows the crude and adjusted relative risk ratios (RRR) associated with individual and couples’ HCT uptake compared to “never tested” among this sub-group of 10,712 individuals.

**Table 3:** Unadjusted* and adjusted relative risk ratios (RRR) of HCT uptake among married individuals observed for at least three study visits between 2003 and 2009

**Characteristic**	Individual HCT**				Couples’ HCT**			
	**N = 10712**	**%**	**Crude Relative Risk Ratios(RRR) [95% Confidence Interval (CI)]**	**Adjusted RRR (95% CI)**	**N = 10712**	**%**	**Crude RRR (95% CI)**	**Adjusted RRR (95% CI)**
**Gender**								
Female	5597	57.5	1.00	1.00	5597	36.4	1.00	1.00
Male	5115	52.3	0.55 (0.43, 0.69)	**0.68 (0.51, 0.90)**	5115	37.5	0.62 (0.48, 0.79)	0.79 (0.59, 1.06)
**Age Group**								
15-24	1929	62.9	1.00	1.00	1929	28.0	1.00	1.00
25-34	5453	55.7	1.18, (0.90, 1.55)	1.27 (0.94, 1.72)	5453	37.6	1.79 (1.34, 2.40)	**1.81 (1.32, 2.50)**
35+	3330	49.4	0.75 (0.55, 1.01)	0.81 (0.57, 1.16)	3330	41.1	1.40 (1.01, 1.93)	1.36 (0.93, 1.97)
**Education**								
None	653	58.0	1.00	1.00	653	39.2	1.00	1.00
Primary	7133	54.5	0.33 (0.16, 0.65)	**0.32 (0.16, 0.65)**	7133	37.6	0.33 (0.17, 0.67)	**0.33 (0.16, 0.68)**
Post-primary	2926	55.7	0.28 (0.14, 0.56)	**0.27 (0.13, 0.54)**	2926	34.8	0.26 (0.13, 0.53)	**0.24 (0.12, 0.50)**
**Non-marital relations in past year**								
No	8387	55.0	1.00	1.00	8387	38.1	1.00	1.00
Yes	2325	55.2	0.59 (0.47, 0.74)	0.81 (0.63, 1.04)	2325	32.9	0.51 (0.40, 0.65)	**0.61 (0.46, 0.79)**
**Prior HCT**								
No	5772	54.5	1.00	1.00	5772	32.8	1.00	1.00
Yes	4940	55.6	4.92 (3.95, 6.12)	**5.12 (4.11, 6.39)**	4940	41.7	6.13 (4.91, 7.65)	**6.80 (5.44, 8.51)**
**Couple HIV status**								
M-F-	9360	54.2	1.00	1.00	9360	37.9	1.00	1.00
M + F+	645	63.9	0.86 (0.66, 1.13)	**0.65 (0.42, 0.98)**	645	24.2	0.88 (0.66, 1.16)	**0.32 (0.12, 0.51)**
M-F+	404	62.9	0.76 (0.58, 0.99)	**2.46 (1.26, 4.80)**	404	33.4	0.84 (0.63, 1.11)	1.69 (0.86, 3.34)
M + F-	303	50.8	0.61 (0.44, 0.86)	0.83 (0.45, 1.53)	303	39.6	0.64 (0.45, 0.91)	0.91 (0.49, 1.69)

*Table includes all variables that were significantly associated with HCT uptake in the bivariate analysis.

**Never tested is used as the base outcome.

#### Predictors for individual HCT uptake

At the bivariate analysis, being male (RRR = 0.55; 95% Confidence Interval (CI): 0.43, 0.69), having primary (RRR = 0.33; 95% CI: 0.16, 0.65) or post-primary education (RRR = 0.28; 95% CI: 0.14, 0.56), reporting non-marital relations in the past year (RRR = 0.59; 95% CI: 0.47, 0.74), being in an HIV-discordant relationship where the female (RRR = 0.76, 95% CI: 0.58, 0.99) or male partner was HIV-positive (RRR = 0.61, 95% CI: 0.44, 0.86) was associated with less likelihood of individual testing (Table 3). Only one factor (prior HCT) was positively associated with individual uptake of HCT with individuals who had prior HCT being 4.9 times more likely to receive individual HCT than those without prior HCT (RRR = 4.92; 95% CI: 3.95, 6.12). After adjusting for potential confounders, including socio-demographics (age-group, gender, education) and behavioral factors (non-marital relations, prior HCT, and couple HIV status), the factors that were significantly associated with less likelihood of individual testing were: being male (Adjusted (Adj.) RRR = 0.68; 95% CI: 0.51, 0.90), having primary (Adj. RRR = 0.32; 95% CI: 0.16, 0.65) or post primary education (Adj. RRR = 0.27; 95% CI: 0.13, 0.54), and being in a concordant HIV-positive marital relationship (Adj. RRR = 0.65; 95% CI: 0.42, 0.98). Being in an HIV-discordant relationship where the female partner was HIV-positive (Adj. RRR = 2.46; 95% CI: 1.26, 4.80) and prior receipt of any form of HCT (Adj. RRR = 5.12; 95% CI: 4.11, 6.39) were positively associated with increased likelihood for individual testing (Table 3).

#### Predictors of couples’ HCT uptake

Bivariate analysis found that being male (RRR = 0.62; 95% CI: 0.48, 0.79), having primary (RRR = 0.33; 95% CI: 0.17, 0.67) or post-primary education (RRR = 0.26; 95% CI: 0.13, 0.53), reporting extra-marital relationships in the past year (RRR = 0.51; 95% CI: 0.40, 0.65) and being in an HIV-discordant relationship where the male partner was HIV-positive (RRR = 0.64; 95% CI: 0.45, 0.91) were associated with less likelihood of couples’ HIV testing (Table 3). The bivariate factors that were positively associated with increased likelihood of couples’ HCT were being 25-34 years of age (RRR = 1.79; 95% CI: 1.34, 2.40), or 35 years and older (RRR = 1.40; 95% CI: 1.01, 1.93) and prior HCT (RRR = 6.13; 95% CI: 4.91, 7.65). After adjusting for potential confounders, the factors that were significantly associated with less likelihood of couples’ HCT were: having primary (Adj. RRR = 0.33; 95% CI: 0.16, 0.68) or post-primary education (Adj. RRR = 0.24; 95% CI: 0.12, 0.50), reporting extra-marital relations in the past year (Adj. RRR = 0.61; 95% CI: 0.46, 0.79), and being in a concordant HIV-positive marital relationship (Adj. RRR = 0.32; 95% CI: 0.12, 0.51). The factors that were associated with increased likelihood of couples’ testing were: age-group 25-34 (Adj. RRR = 1.81; 95% CI: 1.32, 2.50) and prior receipt of HCT (Adj. RRR = 6.80; 95% CI: 5.44, 8.51).

## Discussion

Our study examining the trends in HCT among 21,798 married individuals enrolled in the Rakai Community Cohort Study (RCCS) between 2003-2009 reveals three interesting and important trends: (i) the proportion of never-tested individuals increased significantly over time, (ii) uptake of individual HCT increased between 2003 and 2005 but declined between 2006 and 2009, and (iii) uptake of couples’ HCT remain low and stabilized below 30%. We also found among a sub-set of our study population that prior receipt of HCT was a significant predictor of individual and couples’ HCT, suggesting high levels of repeat testing [27] and the need to devise alternative approaches to create demand for HCT uptake among individuals with no prior HCT.

The finding that the proportion of those who had never tested for HIV increased from 15% in 2003/04 to 18% in 2008/09 (*P* < 0.0001) is worrying given that not knowing one’s HIV status is a major impediment to accessing appropriate HIV prevention, care and treatment [28]. While this study cannot definitively answer why fewer people were testing over time, our regression analysis did find that males, individuals reporting non-marital sexual relationships; and participants with primary or post-primary education, were less likely to test for HIV. Overall males reported higher levels of non-marital relationships (over 40%) and individuals with non-marital relationships may be more fearful of an HIV positive test results. Interestingly, the decline in non-testing among discordant couples is consistent with an increase in individual uptake of HCT among those in HIV-discordant relationships where the female partner was HIV-positive.

In terms of individual HCT, the proportion tested increased from 58% in 2003 to 60% in 2005/06 but then declined to 54% in 2008/09 (P < 0.0001). The decline in individual HCT uptake and the increase in non-testing behaviors noted above may be linked to a change in the HCT delivery strategy. In 2005, the HCT delivery strategy was changed from exclusive home-based HCT to selective home-based delivery of HIV test results and the associated post-result counseling services. After the change in strategy, only first-time testers and couples with at least one HIV-positive partner continued to receive HCT in their homes. All the other participants had to access their HIV test results at the community counseling offices that were located in the study communities. Earlier data from Rakai show that home-based HCT was associated with higher uptake of HCT [23,29]. The change in the HCT delivery strategy may in part account for the increase in the proportion of married individuals who had never tested and the decline in individual HCT rates observed after 2005. However, it is important to note that even with the change of HCT delivery strategy, HCT services continued to be freely available within the study communities, and the community counseling offices continued to exist within easy reach of the study participants. This suggests that the decline in individual HCT and increase in non-testing observed in married individuals might be explained by other reasons which were not documented as part of this study. Further research on why uptake of individual or couples’ HCT did not increase despite the availability of free HCT services in the study communities might help to unravel the actual reasons behind the observed trends.

This study also found that couples HCT remained consistently below 30% over the years despite the provision of free HIV counseling and testing services in the study communities. This proportion represents an increased from the 17% originally reported in this cohort [29], however, this proportion remains low when compared with couples’ HCT uptake rates reported from Bushenyi district of Uganda [13] and in southern Zambia [30]. Our findings suggest that uptake of couples’ HCT was lower in younger couples (15-24 year-old) compared to older couples (25 years and above). This raises serious public health concerns considering that majority of these couples are at an earlier stage of marital formation and would benefit from forming marriages and/or starting a family when both of them are aware of each other’s HIV status. A recent mathematical model on the dynamics of extra-couple HIV transmission (i.e. HIV transmission occurring from outside the primary relationship) in sub-Saharan Africa has found that women have a high infection risk before entering into a cohabiting partnership [31].

We also found that prior HCT was significantly associated with couples’ HCT uptake. This is likely due to the fact that prior HCT can confer confidence and reduce the fears associated with receipt of HCT. Of interest was the finding that couples’ HCT increased significantly in couples where at least one of the partners was HIV-positive, with the greatest increase observed in couples where the female partner was HIV-positive (from 13.9% in 2003/04 to 33.6% in 2008/09, *P* < 0.0001). Driving this finding potentially is the intensified efforts to promote counselor-facilitated HIV status disclosure within the Rakai cohort [32] and also due to increased availability of antiretroviral therapy in Rakai since 2004. It is important to note, however, that among couples in which both partners were HIV-negative, there was a non-significant decrease in the proportion receiving individual HCT over time. The reasons for this apparent decrease in couples’ HCT among HIV-negative concordant partners were not documented as part of this study, necessitating further research to explore these observations.

Overall, the results of this study provide further evidence supporting the need for the development and testing of innovative HIV counseling and testing strategies to create demand for couples’ HCT. Potential approaches include sending out invitations to male partners to test with their female partners at antenatal care clinics where uptake is historically low among males [33] and promotion of couples’ HCT through influential network agents [34,35], an approach yet to be evaluated beyond Rwanda and Zambia. Other potential strategies include targeting partners of index clients enrolled in HIV care [36], and attracting men to test for HIV together with their partners through men-only meetings as well as community-based efforts to sensitize couples about the benefits of couples’ HCT, e.g. through couple-focused meetings.

This study had several limitations. We have indicated that HIV risk perception was assessed from all study participants, including those who had ever tested HIV-positive. There is a possibility that retaining those who were HIV-positive in the final analyses might lower the odds of HIV testing in this population, given that HIV-positive individuals are less likely to test for HIV [23]. However, when the effect of HIV risk perception on HCT uptake was assessed exclusively in HIV-negative individuals, we did not find any significant effect on either individual or couples’ HCT at the bivariate analysis level. Nevertheless, since HIV risk perception has been found to affect individual HCT uptake in other studies [37,38], the effect of risk perception observed in this population should be interpreted with caution. Another limitation is that the trends observed within the cohort might be a result of self-selection, since our findings show that those who had prior HCT were more likely to have tested again; and there is a possibility that uptake of individual or couples’ HCT reported in subsequent visits might be a result of repeat testing rather than uptake of HCT among new testers. Since this study did not explore the proportion of repeat vs. new testers, we cannot determine the direction of this limitation. It should be noted that the findings presented in this article are based on the number of married individuals who had complete HIV status and HCT receipt information at each study visit, and may therefore not be representative of all married individuals, since some couples may have opted not to participate in all the study visits, or participated but did not have complete HIV status and HCT information available at the time of analysis. However, since the RCCS is an open-cohort study, there is a possibility that the number of married individuals enrolled at each visit might be representative of all married individuals in the study communities at the time.

Despite these limitations, this study is unique in that it uses population-based data from a large, ongoing community cohort study (RCCS) that has followed up study participants annually since 1994. This presents a greater opportunity for assessing long-term trends in the same communities and understanding HCT dynamics among married couples in the general population context. It also reflects a significant shift away from studies based at specialized HIV clinics or among specific population sub-groups, such as women accessing PMTCT services. Another unique aspect of this study is that it was implemented in an area where HCT is provided in the communities free of charge and all participants who test HIV-positive are immediately linked to HIV care and treatment. These conditions are favorable for increased uptake of HCT, including couples’ HCT. Despite these conditions, uptake of couples’ HCT remained low, pointing to the challenges inherent in increasing uptake of couples’ HCT beyond access issues. The recent implementation of innovative HCT promotional approaches, including use of community-based influential network agents [35] might be one of the key strategies necessary to improve couples’ HCT uptake in sub-Saharan Africa.

## Conclusion

The proportion of married individuals that received couples’ HCT remained low (below 30%) over the years, while uptake of individual HCT declined over time. Prior receipt of HCT was a significant predictor of both individual and couples’ HCT uptake. These findings call for innovative strategies to create demand for couples’ HCT, especially among couples with no prior HCT experience.

## Competing interests

The authors declare that they have no competing interests.

## Authors’ contributions

JKBM conceived the study, prepared the data analysis plan, interpreted the data and wrote the first draft of this paper. JS & FM analyzed the data and contributed to interpretation of the data. JD, EO, NM, RKW & DS contributed to the interpretation of data and revised the paper for substantial intellectual content. All authors read and approved the final manuscript.

## Pre-publication history

The pre-publication history for this paper can be accessed here:

http://www.biomedcentral.com/1471-2458/13/618/prepub
