# Erratum to: Trends in HIV counseling and testing uptake among married individuals in Rakai, Uganda

**DOI:** 10.1186/s12889-017-4228-5

**Published:** 2017-04-18

**Authors:** Joseph K. B. Matovu, Julie Denison, Rhoda K. Wanyenze, Joseph Ssekasanvu, Fredrick Makumbi, Emilio Ovuga, Nuala McGrath, David Serwadda

**Affiliations:** 1 School of Public Health, Makerere University College of Health Sciences, P.O. Box 7072, Kampala, Uganda; 2 Johns Hopkins Bloomberg School of Public Health, Baltimore, MD USA; 3 Rakai Health Sciences Program/Uganda Virus Research Institute, P.O. Box 49, Entebbe, Uganda; 4 Gulu University, P.O. Box 166, Gulu, Uganda; 5 University of Southampton, Southampton, UK

## Erratum

Following publication of this article [1], it has come to our attention that the total number of observations (21,798) has been mistakenly cited as the number of respondents in some paragraphs within the paper. This number appears within the abstract, in the analysis sub-section, within the opening paragraph of the results section, and in the section on HIV prevalence. The number that should have been cited is 11,268 - the total number of respondents in the dataset. 21,798 refers to the total number of observations over the study period. The percentages estimated out of 21,798 have been recalculated. Of the 11,268 individuals enrolled in this study, 81.2% (9,220) were in monogamous marital unions while 18.2% (2,048) were in polygamous marital unions. Of those in polygamous marital unions (n = 2,048), 52.8% were females while 47.2% were males. Thirty eight per cent of the participants (4,236) reported that they had ever received HCT (i.e. individual or couples’ HCT). Overall HIV prevalence was 11.9% (1,337 of 11,268). However, it is important to note that since serial cross-sectional analyses of each of the 4 study visits were used under consideration, the findings shown in Tables 1 and 2 as well as Fig. 2 (A,B,C) are not affected by this error.

In Table 3, percentages are cited showing the number of individuals who were interviewed for at least 3 times. The percentages are shown against their denominators. In citing the numbers, the total number of observations (10,712) was used for those that were interviewed for at least 3 times, instead of 4338 - which is the total number of individuals interviewed for at least 3 times. The corrected version of the table is provided below.

**Table 3:** Unadjusted and Adjusted Relative Risk Ratios (RRR) of prior receipt of HCT among 4338 married or cohabiting individuals who participated in the RCCS in at least three study visits between 2003 and 2009 (total observations: 10,712)

Characteristic^a^	Individual HCT^b^				Couples’ HCT^b^			
	*N* = 4338	%	Unadjusted Relative Risk Ratios (RRR) [95% Confidence Interval (CI)]	Adjusted RRR (95% CI)	*N* = 4338	%	Unadjusted RRR (95% CI)	Adjusted RRR (95% CI)
Sex								
Female	2287	52.7	1.00	1.00	2287	52.7	1.00	1.00
Male	2051	47.3	0.55 (0.43, 0.69)	0.68 (0.51, 0.90)	2051	47.3	0.62 (0.48, 0.79)	0.79 (0.59, 1.06)
Age Group								
15–24	414	9.5	1.00	1.00	414	9.5	1.00	1.00
25–34	2227	51.3	1.18, (0.90, 1.55)	1.27 (0.94, 1.72)	2227	51.3	1.79 (1.34, 2.40)	1.81 (1.32, 2.50)
35+	1697	39.1	0.75 (0.55, 1.01)	0.81 (0.57, 1.16)	1697	39.1	1.40 (1.01, 1.93)	1.36 (0.93, 1.97)
Education								
None	635	5.6	1.00	1.00	635	5.6	1.00	1.00
Primary	7376	65.5	0.33 (0.16, 0.65)	0.32 (0.16, 0.65)	7376	65.5	0.33 (0.17, 0.67)	0.33 (0.16, 0.68)
Post-primary	3257	28.9	0.28 (0.14, 0.56)	0.27 (0.13, 0.54)	3257	28.9	0.26 (0.13, 0.53)	0.24 (0.12, 0.50)
Non-marital relations in past year								
No	8558	75.9	1.00	1.00	8558	75.9	1.00	1.00
Yes	2710	24.1	0.59 (0.47, 0.74)	0.81 (0.63, 1.04)	2710	24.1	0.51 (0.40, 0.65)	0.61 (0.46, 0.79)
Previous HCT								
No	7032	62.4	1.00	1.00	7032	62.4	1.00	1.00
Yes	4236	37.6	4.92 (3.95, 6.12)	5.12 (4.11, 6.39)	4236	37.6	6.13 (4.91, 7.65)	6.80 (5.44, 8.51)
Couple HIV status								
M-F-	9418	83.6	1.00	1.00	9418	83.6	1.00	1.00
M + F+	848	7.5	0.86 (0.66, 1.13)	0.65 (0.42, 0.98)	848	7.5	0.88 (0.66, 1.16)	0.32 (0.12, 0.51)
M-F+	506	4.5	0.76 (0.58, 0.99)	2.46 (1.26, 4.80)	506	4.5	0.84 (0.63, 1.11)	1.69 (0.86, 3.34)
M + F-	496	4.4	0.61 (0.44, 0.86)	0.83 (0.45, 1.53)	496	4.4	0.64 (0.45, 0.91)	0.91 (0.49, 1.69)

^a^Table includes all variables that were significantly associated with prior receipt of HCT in the bivariate analysis. ^b^Never tested is used as the base outcome
